# Retreatment of Experimental Carrier-Based Obturators with the Remover NiTi Instrument: Evaluation of Apical Extrusion and Effects of New Kinematics

**DOI:** 10.1155/2021/2755680

**Published:** 2021-10-23

**Authors:** Chiara Pirani, Francesco Iacono, Fausto Zamparini, Luigi Generali, Carlo Prati

**Affiliations:** ^1^Endodontic Clinical Section, School of Dentistry, Department of Biomedical and Neuromotor Sciences (DIBINEM), Alma Mater Studiorum University of Bologna, Bologna, Italy; ^2^Department of Surgery Medicine, Dentistry and Morphological Sciences with Transplant Surgery, Oncology and Regenerative Medicine Relevance (CHIMOMO), University of Modena and Reggio Emilia, Modena, Italy

## Abstract

The objective of this study is to evaluate the retreatment time and weight of apically extruded debris yielded by two different kinematics during the removal of different root canal filling materials. Forty straight single-rooted extracted teeth were instrumented with HyFlex CM files and obturated with two different techniques: 25.04 HyFlex experimental carrier-based obturators (Coltène/Whaledent, Altstätten, Switzerland) (group 1) or 25.04 single gutta-percha cones (Roeko Coltène/Whaledent, Altstätten, Switzerland) (group 2) and Guttaflow Bioseal as the sealer. Samples were divided into four subgroups (*n* = 10) according to the used kinematics for the removal of root canal filling materials: continuous rotation (A) or retreatment motion (B) with a Remover and HyFlex EDM Nickel-Titanium instruments activated with a CanalPro Jeni micromotor (Coltène/Whaledent, Altstätten, Switzerland). Time for retreatment was digitally recorded, and debris extruded from the apex was collected in Eppendorf tubes and weighted with an analytical balance. Data on retreatment time and apical extrusion were statistically analyzed with the Kruskal–Wallis test (*p* < 0.05). Working length was achieved in all the retreated samples. The removal of root filling material resulted significantly faster with the Jeni mode (*p* < 0.001), and the difference was significant for the removal of both filling materials (*p* < 0.05). No significant differences on debris extrusion between single cone and experimental obturators groups were noted (*p* > 0.05), and no significant differences between kinematics (continuous rotation vs. Jeni motion) were observed (*p* > 0.05). All the tested retreatment strategies lead to an extrusion of material from the apex, and the weight of apically extruded debris was similar. The use of the innovative CanalPro Jeni kinematics accelerates the time for the removal of root filling materials.

## 1. Introduction 

Endodontic orthograde retreatment should be considered as a first approach in failing endodontic cases [1]. Intricate and aberrate anatomies [2] and complex operative procedures [3, 4] make retreatments challenging. Current trends in endodontics are developing instruments and strategies conceived with the aim to simplify the reshaping phase among the complexity of secondary root canal treatments.

One of the major drawbacks during the reshaping phase is the tissues injury of chemical, mechanical, or microbiological nature, most commonly due to the apical extrusion of infected debris and irritants and is also reported as a possible aetiology of postoperative pain [5]. This undesirable consequence is caused by many factors related to the experience of the operators and to the instruments or kinematics utilized. Apical extrusion, which can be associated with induction of inflammation and delayed periapical healing, has been widely investigated by numerous in vitro studies [6–8]. Several studies produced opposing findings on the amount of dentinal debris thrusted through the apical foramen by single-file techniques [9, 10].

In 2020, a new instrument designed for the removal of intracanal root filling materials was introduced (Remover, Coltène/Whaledent, Altstätten, Switzerland). This file consists in a 30.07 single instrument with a variable offset blade, a noncutting tip, and a triple helix section. The Remover Nickel-Titanium (NiTi) file has been treated with a patented heat process (C. Wire) to grant an improved flexibility and an enhanced shape memory that renders the instrument prebendable [11, 12].

Moreover, different strategies of motion have been proposed to enhance the performances of NiTi rotary instruments [13]. A new endodontic motor has been recently marketed, with some unique features (CanalPro Jeni, Coltène/Whaledent, Altstätten, Switzerland) with the aim to minimize the risk of file breakage [14].

In 2021, new experimental carrier-based obturators (Coltène/Whaledent, Altstätten, Switzerland) were designed consisting of a core material composed by a combination of different resins and some organic/inorganic fillers and coated with flowable gutta-percha, with the claimed possibility to be used in combination with GuttaFlow Bioseal (Coltène/Whaledent, Altstätten, Switzerland).

This in vitro study aimed to evaluate the effectiveness of two different kinematics on the removal of two different root canal filling materials. Time to retreatment and apical extrusion of debris generated during retreatment procedures by two different kinematics were investigated. The null hypothesis tested was that there are no significant differences in terms of time to retreatment and apical extrusion between the tested variables.

## 2. Materials and Methods

### 2.1. Selection of Samples

Forty human teeth extracted for orthodontic or periodontal reasons (Ethical Committee Approval no. 0000832) were stored in distilled water at 4°C and selected using the following criteria: single straight canal (<20° angle of curvature) without root canal treatment, completely formed apex, and the absence of canal calcifications. Crowns were removed through a high-speed water-cooled handpiece with a diamond cylindrical bur (Intensiv SA, Montagnola, Swiss), at 15.0 mm from the apex measured with an endodontic measuring gauge (Dentsply Maillefer, Ballaigues, Swiss). A preoperative digital X-ray of each root was performed in buccolingual and mesiodistal directions to ensure standardization of the selected samples.

A previous study [10] was used to identify an effect size of 0.64 required to calculate the total sample size for this study error = 0.05, and power (1−) = 0.90 was also input. A total of 40 samples were indicated as the minimum to observe differences between systems (F-test family, ANOVA, G*∗*Power for Mac).

### 2.2. First Root Canal Treatment

A 25 mm #10 stainless steel manual K-file (Dentsply Maillefer, Ballaigues, Swiss) was used to assess initial patency and to evaluate the working length (WL). Coronal thirds were enlarged with the 25.08 HyFlex CM (Coltène/Whaledent, Altstätten, Switzerland) orifice opener followed by K-file #10–15 and HyFlex CM 20.04, 25.04, 20.06 at WL following the manufacturers' recommendations at 500 rpm and 2.5 Ncm in continuous rotation. Each canal was irrigated with 5 ml of 5.25% NaOCl (Niclor 5 Ogna, Muggiò, Italy) and 3 ml of 10% EDTA (Tubuliclean Ogna, Muggiò, Italy) solutions during instrumentation. Each canal was subjected to a further irrigation with 1.0 ml of 5.25% NaOCl for 3 minutes, 0.5 ml 10% EDTA for 1 minute, and 3 minutes with 1.0 ml of NaOCl [15]. Final irrigation was performed with sterile water, and canals were dried with sterile paper points (Mynol, Milwaukee, WI).

Samples were randomly divided into 2 groups, according to the obturation method.

  Group 1: size 25.04 experimental carrier-based obturators (Coltène/Whaledent, Altstätten, Switzerland) and Guttaflow Bioseal (lot. N. K41983) as the sealer were used. Guttaflow Bioseal was homogeneously mixed at a ratio of 1 : 1 by means of an applicator with a syringe mixing tip (Coltène/Whaledent, Altstätten, Switzerland). A thin layer of sealer was introduced on the canal walls using the last sterile K-file dimension used apically until the WL, and the excess were removed with another sterile matching paper point according to the manufacturers' recommendations. Experimental carrier-based obturators (Coltène, Ohio, USA) were heated using the Herofill Oven (Micro-Mega, Besançon, France) for the recommended time until the indicator emitted a sound. After heating, obturators were slowly inserted into the canal until reaching the WL, with firm and steady pressure. Obturators were separated after 120 s, and a heated plugger instrument was used to compact the material only at the entrance of the canal.
  Group 2: single 25.04 gutta-percha cones (Roeko Coltène/Whaledent, Altstätten, Switzerland) customized for each sample to obtain adequate tug-back at WL were used to fill the canals as control, with Guttaflow Bioseal as the sealer.

A coronal seal was obtained with a temporary filling (Coltosol F, Coltène/Whaledent, Altstätten, Switzerland) in every samples, and a periapical radiograph was acquired in mesiodistal and buccolingual angulations to verify the filling quality. Samples were then stored in 15 ml Hank's Balance Salt Solution (HBSS, Lonza, Verviers, Belgium) used as the simulated body solution in plastic containers, for 30 days at 37°C and 100% humidity. Two trained endodontists performed all the treatment and retreatment procedures.

### 2.3. Retreatment Procedure with the Remover

All samples were randomly assigned to 4 subgroups (*n* = 10 each) according to the retreatment procedure tested. Tested groups are summarized in Table 1.

  Group 1A−2A : HyFlex EDM 25.12 orifice opener was used to drill a pilot hole of 2-3 mm inside the gutta-percha obturator. The Remover instrument 30.07 (Coltène/Whaledent, Altstätten, Switzerland) (lot. N. 612861) in continuous rotation with an endodontic CanalPro Jeni micromotor (Coltène/Whaledent, Altstätten, Switzerland) was used at a constant speed of 800 rpm and 2.5 Ncm. The Remover was guided to progress inside the filling material in the coronal and middle third since 3 mm from the WL with back-and-forth motions of 2-3 mm without apical pressure, following manufacturers' recommendations. Apical third was prepared until WL with HyFlex EDM One File 25.08 and 40.04 instruments (Coltène/Whaledent, Altstätten, Switzerland) used in continuous rotation at 500 rpm and 2.5 Ncm. During every retreatment procedure, every 4 strokes, the instrument was pulled out from the canal and the material entrapped among the spires was displaced using a sterile sponge. When the instrument reached the WL and no debris was observed between the spires, the retreatment procedure was considered completed. During removal of the root canal filling material, 10 mL of distilled water was used to irrigate canals by using 27-gauge side-vended needles.
  Group 1B–2B: the same protocol for the Remover and HyFlex EDM instruments activated with a CanalPro Jeni micromotor (Coltène/Whaledent, Altstätten, Switzerland) was used with the selected “Retreatment” motion. A gentle and continuous pressure to the file was applied until the acoustic signal; therefore, the file was removed from the canal and irrigation was performed. The preparation was carried on with the same instrument until the automatic acoustic signal indicated that the file should be replaced.

No solvent was used. A digital radiograph of each retreated root was taken in mesiodistal and buccolingual angulations to evaluate the presence of residual filling material.

### 2.4. Retreatment Time Evaluation

Total time for retreatment was digitally recorded in seconds including active instrumentation, instrument changes, irrigation, and spires checking and cleaning. Data were statistically analyzed. Incidence of instrument fracture was recorded.

### 2.5. Apical Extrusion of Debris

**Figure 1:**
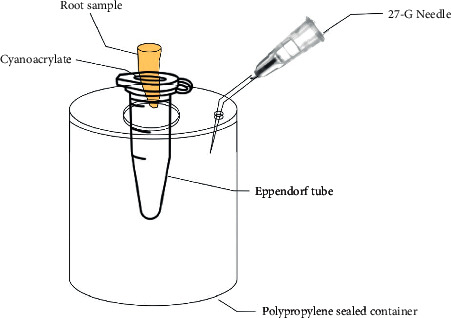
A representative schematic drawing of the experimental apparatus used to collect debris extruded from the apex during retreatment procedures.

Each sample was inserted to its cementoenamel junction (CEJ) in a hole created in the cap of an Eppendorf tube, which had been preweighed 3 times using an analytical balance (Bel Engineering series M, Monza, Italy) with an accuracy of 10^–5^ g to collect apically extruded debris. The tooth was fixed to the CEJ using cyanoacrylate (Rocket, DVA, Corona, CA USA). The apical part of the root was suspended within the tube, which acted as a collecting container for the material extruded by the foramen of the root. A 27-G needle was placed through the rubber stopper to equalize the air pressure inside and outside the vial [16]. The system composed by a cap, tooth, and needle was applied to its Eppendorf tube, and the tubes were fitted into polypropylene sealed containers to prevent the operator from viewing debris extrusion during the experimental process (Figure 1). The entire apparatus was handled only by the vial. After instrumentation, the cap, needle, and tooth were removed from the Eppendorf tube and the debris attached to the root surface was collected by washing the root with 1 mL distilled water whilst in the tube. Tubes were stored at 68°C for 5 days in an incubator to evaporate the distilled water, and the weight calculation was blindly performed to the group assignment. The Eppendorf tubes containing the extruded debris were weighed 3 times to obtain the final weights of the tubes, and the mean value was obtained [17]. The amount of the extruded debris was calculated by subtracting the weight of the first calculation from the weight of the dry tube.

### 2.6. Statistical Analysis

Statistical analysis was performed with SPSS statistics software (version 23.0, Chicago, IL, USA). Given the non-Gaussian distribution (normality test >0.05), data were statistically analyzed using the nonparametric Kruskal–Wallis test on independent samples to identify statistically significant differences. Time for material removal and apical extrusion of debris were analyzed considering the different kinematics and the different obturation materials. The level of significance was set at *p*=0.05.

## 3. Results

**Figure 2:**
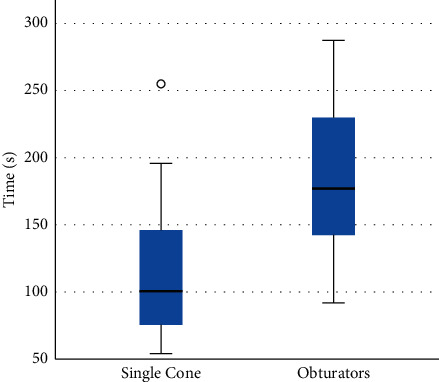
Mean values of retreatment time (sec) measured for each obturation material group. In each box plot, the median value (black line), interquartile range (length of the box), and minimum and maximum values (extreme lines) are reported. °indicates outlier values. No significant differences are reported.

**Figure 3:**
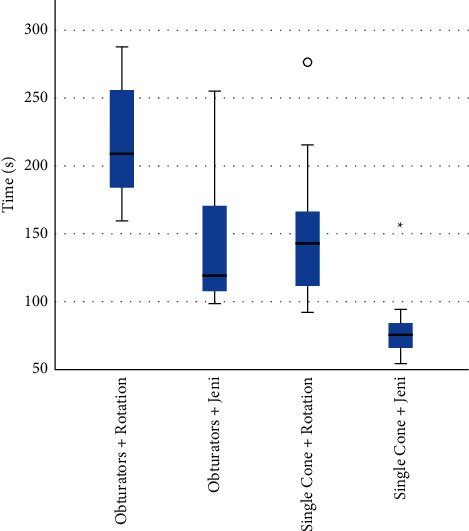
Mean values of retreatment time (sec) measured for each experimental group (kinematics). In each box plot, the median value (black line), interquartile range (length of the box), and minimum and maximum values (extreme lines) are reported. °indicates outlier values. Significant differences are reported (^∗^).

The WL was achieved in all the retreated samples. Time for root filling removal expressed in seconds (s) is summarized in Table 2. The removal of root filling material resulted significantly faster with the Jeni mode than with continuous rotation (*p* > 0.001). The difference was significant for the removal of both filling materials (*p* < 0.05). Removal with the Jeni motion was significantly faster than with continuous rotation in group 1 (*p*=0.040) and in group 2 (*p*=0.010). Removal of obturators with continuous rotation resulted significantly slower than for single cone and resulted as follows: group 1A > group 1B = group 2A > group 2B (*p* < 0.05) (Figures 2–3).

During retreatment procedures, one Remover instrument fractured (group 1A) and apical microcracks were observed in 3 samples (1B, 2A, and 2B groups/one sample each). Postoperative radiographs revealed the presence of residual filling material independently by the kinematics and obturation technique. The amount of remaining material was mainly located in the coronal third (33%), followed by the middle third (28%) and apical third (10%).

**Figure 4:**
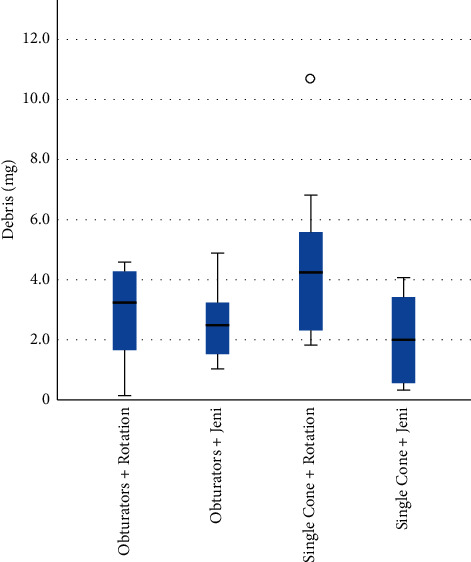
Mean values of debris apical extrusion (grams) measured for each experimental group (kinematics). In each box plot, the median value (black line), interquartile range (length of the box), and minimum and maximum values (extreme lines) are reported. °indicates outlier values. No significant differences are reported.

Mean values and standard deviations (SDs) of apically extruded debris weights are reported in Table 2. A greater weight of apically extruded debris was produced in group 2A, but no significant difference between group 1 and group 2 was noted (*p* > 0.05) and no significant difference between different kinematics (group A vs. group B) were observed (*p* > 0.05) (Figure 4).

## 4. Discussion

This in vitro study aimed to evaluate the weight of debris extruded toward the apex during the removal of the experimental carrier-based system using NiTi systems activated by two different kinematics. To the best of our knowledge, to date, no study evaluated the effectiveness of those different kinematics in association with the Remover file.

One of the primary challenges during retreatments is the removal of previous contaminated filling material allowing instruments and irrigant solutions to act throughout the entire canal space [18]. At the same time, it is fundamental to avoid an undesirable extrusion of debris in the periradicular tissues that can be associated with the induction of inflammation and delayed periapical healing [19, 20]. Furthermore, apical extrusion is one of the most important factors associated with postoperative pain that seems to be related to the instrumentation technique [21]; hence, its evaluation is considered relevant from a clinical point of view.

Every instrumentation strategy leads to an extrusion of material from the apex, and the amount of extruded debris can be varied by kinematics, cross-sectional design, taper, and wire of the NiTi files [17, 22, 23]. Remover is a single-use 30.07 instrument manufactured in C. Wire, and this alloy that underwent thermal treatment has been demonstrated to enhance metallurgical characteristics of conventional NiTi [12]. The higher flexibility provided by the thermomechanically induced martensitic phase [24, 25] may increase the deformation capacity and the ductility of the instruments that could be helpful during their clinical use [26–28]. In the present laboratory study, this instrument was intentionally used to reshape 3-4 canals to simulate clinical conditions of a multirooted tooth.

The size 25 ISO tip is the most commonly used size during instrumentation [29], and for this reason, the final size after retreatment procedure would theoretically incorporate the previous one over the length of the apical canal [18]. Larger apical preparations are justified in failed treatments undergoing further interventions to optimize root canal disinfection, without weakening the root structure. Therefore, in this study, a 30.07 was used to increase the width of canal space in coronal and medium thirds, and the final apical preparation size was completed with 40.04. This apical enlargement resulted in an acceptable apical preparation in all groups, confirmed by the postoperative radiographs that showed the absence of residual material in 90% of the retreated samples. On the other hand, the higher amount of residual filling in the coronal portion (30%) should be cautiously considered in a clinical setting where the use of magnifications and ultrasonic tips can help to enhance the dentinal surface cleanliness. Interestingly, the distribution of residual filling was independent by the kinematics and obturation technique.

Selected samples of the present study were similar for the type of teeth, length of the canal, and root curvature to increase the probability that apical extrusion of debris was related to instrumentation and not to root morphology.

In the present research, Guttaflow Bioseal, a polysyloxane-gutta-percha calcium silicate-bioglass-containing root canal sealer [30], was associated to experimental obturators due to their complementarity expressed by the manufacturer. Recent studies, conducted with different methodologies (micro-CT and confocal laser scanning microscopy), confirmed the good results of the sealer in terms of root filling quality [31], tubule penetration [32], and retreatability with rotary instrumentation [33]. In the current investigation, the WL was reached in all the retreated samples, independently by the kinematics. Moreover, all the tested motions were associated to debris extrusion, and the differences between the quantity of extruded debris were not relevant. The null hypothesis was, therefore, accepted. However, even if the statistical significance was not reached, the use of the Jeni mode tended to cause less extrusion of debris. Further studies should evaluate the chemical composition of debris, due to the relevant impact that different components and materials may have on periapical inflammation [19]. Considering that no other studies have investigated the impact of the Jeni mode on the apical extrusion, the only comparison can be made with the results on adaptive motion. Karataş et al. [34] reported no significant difference between the adaptive and rotational movement, and our findings are in accordance with their conclusions, even with the limitations due to the different tested kinematics.

An interesting result of the current study is that significant differences exist in terms of time for retreatment when the experimental kinematics was used. In fact, the removal of root filling material resulted significantly faster with the Jeni mode (retreatment mode) than with continuous rotation. The CanalPro Jeni motor continuously measures parameters such as pressure, torque, tension, or electrical intensity to weigh out file stresses and adapts its motion in rotating, controlled by algorithms that regulate the rotary movements as well as speeds via feedback of current intensity, torque, and file stress (CanalPro Jeni, Coltène Brochure). Instrumentation in the Jeni mode should be made with continuous pressure, without additional pecking or brushing movements as the motor automatically adjusts its movement. Further studies should deeply evaluate the safety and efficiency of the Jeni mode during shaping procedures. The effect of groundbreaking kinematics in the presence of complex anatomies and different instrumentation strategies deserves to be the object of preclinical studies with the aim to improve daily clinical endodontics.

## 5. Conclusions

The present laboratory study confirmed that all the tested motions generate apical extrusion of debris, with a tendency to a greater debris extrusion with the conventional rotary kinematics compared to the Jeni motion. The use of the innovative Jeni kinematics accelerates the time for the removal of filling materials.

## Figures and Tables

**Table 1:** Summary of the tested groups according to the obturation technique and to the retreatment kinematics.

Obturation technique	Retreatment kinematics	Groups (*n* = 10)
Experimental obturators	Continuous rotation	Group 1A
	Jeni mode	Group 1B

Single cone	Continuous rotation	Group 2A
	Jeni mode	Group 2B

**Table 2:** Weight of apically extruded debris expressed in milligrams (mg) (mean ± standard deviation (SD)) and time expressed in seconds (s) spent for the removal of the root canal filling materials with different kinematics. Different superscript letters in the same column indicate significant differences among groups (*p* < 0.05).

Obturation technique	Time (s)	Retreatment kinematics	Time (s)	Weight of apically extruded debris (mg)
				Mean ± SD
Experimental obturators	182	Continuous rotation	219c	2.81 ± 1.50a
		Jeni mode	144a	2.60 ± 1.22a

Single cone	118	Continuous rotation	153a	4.58 ± 2.75a
		Jeni mode	82b	2.00 ± 1.43a

## Data Availability

The data used to support the findings of this study are included within the article.
